# Planning and Reporting Effective Web-Based RAND/UCLA Appropriateness Method Panels: Literature Review and Preliminary Recommendations

**DOI:** 10.2196/33898

**Published:** 2022-08-26

**Authors:** Jordan B Sparks, Mandi L Klamerus, Tanner J Caverly, Sarah E Skurla, Timothy P Hofer, Eve A Kerr, Steven J Bernstein, Laura J Damschroder

**Affiliations:** 1 VA Center for Clinical Management Research Ann Arbor, MI United States; 2 Department of Internal Medicine University of Michigan Ann Arbor, MI United States; 3 Institute for Healthcare Policy and Innovation University of Michigan Ann Arbor, MI United States; 4 Department of Learning Health Sciences University of Michigan Ann Arbor, MI United States; 5 VA Quality Enhancement Research Initiative (QUERI) Personalizing Options through Veteran Engagement (PROVE) Program Ann Arbor, MI United States

**Keywords:** quality indicators, health care, web-based, virtual, RAND/UCLA appropriateness method, research design, de-implementation, digital health, health research, virtual health research, health technology, researchers, medical professionals

## Abstract

**Background:**

The RAND/UCLA Appropriateness Method (RAM), a variant of the Delphi Method, was developed to synthesize existing evidence and elicit the clinical judgement of medical experts on the appropriate treatment of specific clinical presentations. Technological advances now allow researchers to conduct expert panels on the internet, offering a cost-effective and convenient alternative to the traditional RAM. For example, the Department of Veterans Affairs recently used a web-based RAM to validate clinical recommendations for de-intensifying routine primary care services. A substantial literature describes and tests various aspects of the traditional RAM in health research; yet we know comparatively less about how researchers implement web-based expert panels.

**Objective:**

The objectives of this study are twofold: (1) to understand how the web-based RAM process is currently used and reported in health research and (2) to provide preliminary reporting guidance for researchers to improve the transparency and reproducibility of reporting practices.

**Methods:**

The PubMed database was searched to identify studies published between 2009 and 2019 that used a web-based RAM to measure the appropriateness of medical care. Methodological data from each article were abstracted. The following categories were assessed: composition and characteristics of the web-based expert panels, characteristics of panel procedures, results, and panel satisfaction and engagement.

**Results:**

Of the 12 studies meeting the eligibility criteria and reviewed, only 42% (5/12) implemented the full RAM process with the remaining studies opting for a partial approach. Among those studies reporting, the median number of participants at first rating was 42. While 92% (11/12) of studies involved clinicians, 50% (6/12) involved multiple stakeholder types. Our review revealed that the studies failed to report on critical aspects of the RAM process. For example, no studies reported response rates with the denominator of previous rounds, 42% (5/12) did not provide panelists with feedback between rating periods, 50% (6/12) either did not have or did not report on the panel discussion period, and 25% (3/12) did not report on quality measures to assess aspects of the panel process (eg, satisfaction with the process).

**Conclusions:**

Conducting web-based RAM panels will continue to be an appealing option for researchers seeking a safe, efficient, and democratic process of expert agreement. Our literature review uncovered inconsistent reporting frameworks and insufficient detail to evaluate study outcomes. We provide preliminary recommendations for reporting that are both timely and important for producing replicable, high-quality findings. The need for reporting standards is especially critical given that more people may prefer to participate in web-based rather than in-person panels due to the ongoing COVID-19 pandemic.

## Introduction

The RAND/UCLA Appropriateness Method (RAM), a variant of the Delphi Method, was developed to synthesize existing evidence and the clinical judgement of a panel of medical experts. The goal of this method is to produce recommendations for appropriate treatment of specific clinical presentations, given current best evidence [1]. This method has been widely used to develop care recommendations and performance measures that define quality of care [2-6]; it provides a transparent and systematic approach that can garner trust and acceptance among physicians, other clinicians, patients, payers, and health systems [7].

The RAM classically involves engaging credible experts to evaluate specific clinical presentations in a 2-round rating process. In the initial round, experts independently rate each clinical scenario. During the second round, panelists participate in a 1 to 2-day in-person session where they have an opportunity to review and discuss each other’s first round ratings, revise the initial list of scenarios, and individually rerate each clinical indication. Indications are categorized as “appropriate,” “uncertain,” or “inappropriate” based on panelists’ median score and level of disagreement [1]. Compared to the standard Delphi Method, the RAM does not require panelists to reach group consensus after multiple rating rounds [1,8].

A difficulty in convening appropriate experts in person is their often-limited time and capacity to participate. Thus, there is a need to identify best practices for conducting expert panels via the internet not only to lower barriers to experts’ participation but also to reduce the costs involved with implementing traditional in-person RAMs. While the use of RAMs with a web-based component in health research was increasing prior to COVID-19, the pandemic has greatly accelerated the need for web-based alternatives with improved technology and end-user familiarity with these tools.

While there is a substantial body of literature describing and testing various aspects of the traditional in-person or hybrid RAMs, few studies report using a completely web-based RAM, and even fewer provide detailed descriptions on how the expert panels were conducted. There is often little information or guidance for designing approaches to meet the goals of specific studies. Boulkedid et al [9] published a systematic review of 80 articles published through 2009, finding that 63% used a “modified” Delphi Method but lacked enough detail to replicate or judge the quality of modified approaches to developing recommendations for quality health care indicators. Moreover, measures of process quality, such as consistent panelist engagement, are rarely reported. Because best practices for conducting virtual RAMs are unclear and reporting is inconsistent, we conducted a literature review to develop preliminary recommendations for implementing and reporting virtual RAMs.

## Methods

### Literature Search and Data Abstraction

In March 2019, we searched PubMed to identify studies published from 2009 to 2019 that reported using a virtual RAM to measure the appropriateness of medical care. The following search terms were used to identify relevant articles: “RAND/UCLA Appropriateness Method” OR “RAND Appropriateness Method” OR “Modified RAND” OR “RAND AND panel” AND “online OR e-delphi OR web OR virtual.” The full search strategy can be found in Multimedia Appendix 1. Two reviewers (JS and LD) screened each article and developed a list of inclusion and exclusion criteria, which are described in Multimedia Appendix 2. To be included, articles must have used the RAM to measure the appropriateness of medical care or focused on the development of clinical practice guidelines or performance measures. Moreover, the expert panel ratings must have been completed on the internet. Web-based ratings could include a teleconference component. Articles were included even if they did not report a rerate session or discussion period among panelists. Non-English articles and articles published prior to 2009 were excluded. Studies with goals not aimed toward providers (eg, improving support for patient caregivers) were also excluded. Additionally, articles were excluded if they were reviews or summaries of the literature. Relevant panel process data (ie, first author, year published, title, mode of administration, topic, and objectives) from each article included in our review were abstracted in a predefined matrix (Multimedia Appendix 3 [2,10-21]). Team members (JS, MK, and SS) independently abstracted the same sample of articles twice to (1) ensure that the basic data collected have been correctly entered in the spreadsheet and (2) verify that the selection criteria have been appropriately applied.

Subsequently, we expanded the Delphi reporting categories recommended by Boulkedid et al [9], which formed the basis of our article abstraction template. Specifically, we organized descriptions with respect to the following categories adapted from Boulkedid et al [9]: (1) composition and characteristics of the web-based RAM expert panels; (2) characteristics of the web-based RAM panel procedures; (3) results; and (4) panel satisfaction and engagement. Additional information abstracted into the matrix included the following: (1) descriptions of how quality indicators were selected; (2) the method used for participant recruitment; (3) whether materials were sent to participants prior to the first expert panel rating; (4) Delphi panel size and composition, as well as the duration of time the panel was convened; (5) purpose of convening the RAM panels; (6) criteria used to rate indications; (7) the web-based system used to host the panels; (8) the number of reported Delphi rounds; (9) description of feedback provided to panelists; and (10) descriptions of participation levels in the discussion rounds. Additionally, we captured information about second or third rounds of ratings and how the final list of indications was selected. Lastly, we captured information about panelist satisfaction and engagement. Four authors (JS, LD, MK, and SS) completed a second, more detailed, data abstraction. The authors independently reviewed each article and completed several rounds of data verification.

### Ethics Approval

The ASSURES study was approved by the Ann Arbor VA Healthcare System IRB (project ID: 1597260).

## Results

### Article Selection

**Figure 1:**
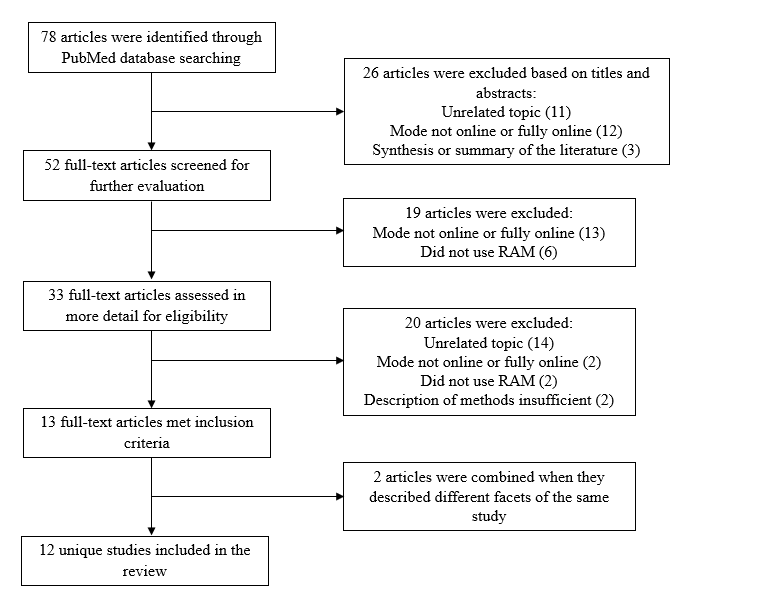
Article search and selection flowchart. RAM: RAND/UCLA Appropriateness Method.

We identified 78 articles that reported using an “online” or “virtual” RAM from our narrative review; 26/78 (33%) articles were excluded based on the title or abstract (Figure 1). A full-text review of 52/78 (67%) articles was completed, resulting in the exclusion of an additional 39/52 (75%) articles; 13/52 (25%) articles were included in the review. We combined 2 published articles that met the inclusion criteria but described different facets of the same study, so the final review included 12/52 (23%) unique studies. Subsequent calculations are based on these 12 studies. The studies included in this literature review used completely web-based RAM approaches to accomplish their goals that ranged from developing quality performance measures or indicators to setting clinical practice standards. Throughout the manuscript, we use the term “indication” or “indicator” to standardize the description of statements panelists were asked to approve during the RAM process. Based on our narrative review, we developed foundational reporting recommendations from Boulkedid et al [9].

### Recruitment and Composition of Web-Based Expert Panels

Table 1 summarizes the characteristics of the RAM expert panels included in our review [2,10-21]. The methods for identifying and recruiting participants were not reported in 5/12 (42%) studies [13-15,18-20]. Of those who relied on professional networks or a snowball sampling approach, 3 (50%) reported inviting prospective participants via email [10,11,17].

The number of people invited to participate in the panels was reported in 9/12 (75%) of studies, ranging from 20 to 352 people with a median of 50 [2,10-13,16,17,20,21]. Of the 12 studies, 9 (75%) reported the number of panelists who participated in at least the first rating, ranging from 10 to 102 individuals, with a median of 42 [2,10-13,16-21]. In addition, all studies described included the types of stakeholders or experts who participated in the panels. In 11/12 (92%) studies, panelists were clinicians related to the topic studied [2,10-13,16-21]; 5 (42%) also included patients or people living with the condition studied [2,11,12,17,21]. Three-quarters of studies (9/12) had between one and two types of stakeholders. An additional 2 (17%) studies included three types of stakeholders; 1 (8%) study reported four or more types.

**Table 1:** Characteristics of the RAND/UCLA Appropriateness Method panels and procedure included in literature review (N=12).

Characteristics		Values
**Methods for recruiting individuals** **(7/12, 58%), n (%)**		
	Professional networks or stakeholders	6 (86)
People invited to participate (9/12, 75%), median (min-max^a^)		50 (20-352)
Participated in first rating (9/12, 75%), median (min-max)		42 (10-102)
**Type of stakeholders^b^, n (%)**		
	Clinicians^c^	11 (92)
	Patients	5 (42)
	Other	5 (42)
**Stakeholder types per study, n (%)**		
	1	6 (50)
	2	3 (25)
	3	2 (17)
	≥4	1 (8)
**Type of RAND^d^ procedure, n (%)**		
	Partial RAM^e^	7 (58)
	Full RAM	5 (42)
Number of rounds (11/12, 92%), median (min-max)		3 (1-3)
**Topic, n (%)**		
	Performance or outcome assessment measures	6 (50)
	Assessment criteria	2 (17)
	Prescribing indicators	1 (8)
	Documentation standards	1 (8)
	Antibiotic stewardship	1 (8)
	Clinical practice standards	1 (8)
**Web-based platform or system used (9/12, 75%), n (%)**		
	ExpertLens	4 (44)
	SurveyMonkey	3 (33)
	Canadian Fluid Survey System	1 (11)
	REDCap^f^	1 (11)
**Methods used to select indicators for the survey^b^, n (%)**		
	Literature review	8 (67)
	Stakeholder feedback	6 (50)
	Prior surveys	2 (17)
	Focus group	1 (8)
	Other	1 (8)
**Indicators in the first rating, median (min-max)**		48 (6-524)
Prepanel materials, n (%)		1 (8)
Duration of consensus process (weeks), median		12
**Geographical scope, n (%)**		
	National	9 (75)
	International	3 (25)
**Item selection or rating criteria^b^, n (%)**		
	Importance	4 (33)
	Validity	4 (33)
	Relevance	4 (33)
	Feasibility	3 (25)
	Other	3 (25)
	Likelihood of use	2 (17)
	Appropriateness	2 (17)
**Number of selection criteria used, n (%)**		
	1	4 (33)
	2	2 (17)
	3	1 (8)
	4	5 (42)
**Feedback provided after first rating (7/12, 58%), n (%)**		
	Quantitative^g^	4 (57)
	Quantitative and qualitative^h^	2 (29)
	Other	1 (14)
**Discussion process^b^ (6/12, 50%), n (%)**		
	Asynchronous	5 (83)
	Anonymous	5 (83)
	Moderated	5 (83)
**Rating process reported (8/12, 67%), n (%)**		
	Rating 2	7 (88)
	Rating 3	1 (13)
**Item selection process, n (%)**		
	Median score + IPR^i^/IPRAS^j^ consensus	8 (67)
	Median score + percentage of agreement	1 (8)
	Percentage of agreement	1 (8)
	Average score	1 (8)
	Other	1 (8)
Process assessment; satisfaction, n (%)		3 (25)
Limitations noted, n (%)		11 (92)

^a^Min-max: minimum-maximum.

^b^The total percentages may exceed 100% because some studies used more than one criterion.

^c^Clinicians include people who actively work in health care settings such as hospitals and clinics to deliver care to patients (ie, doctors, nurses, pharmacists, etc).

^d^RAND corporation.

^e^RAM: RAND/UCLA Appropriateness Method.

^f^REDCap: Research Electronic Data Capture.

^g^Quantitative: group median, minimum, and maximum ratings. Feedback may include panelists’ own ratings to illustrate position versus group ratings.

^h^Qualitative: abstract of panelists’ comments.

^i^IPR: interpercentile range.

^j^IRPAS: interpercentile range adjusted for symmetry.

### Characteristics of Web-Based Expert Panel Procedures

Table 1 summarizes the RAM procedural characteristics across the 12 studies; 5 (42%) studies described the use of all the steps specified by the RAM method (ie, full RAM) [2,11,12,16,21]; the remaining 7 (58%) studies reported the use of some but not all the steps (ie, partial RAM) [10,13-15,17-20]. All studies reported between one and three rating rounds, with a median of 3 rounds. In 6/12 (50%) studies, the RAM was used to develop a set of performance indicators (ie, indicators of clinical care quality) [2,10,11,13,16,21], and the remaining studies focused on developing indications for documenting or describing specific conditions (eg, rheumatoid arthritis and dental caries) [12,14,15,17-20]. The type of web-based system used to conduct the expert panel was reported in 9 (75%) studies [2,11-13,16,17,19-21]. Of these, 4 (44%) studies used the ExpertLens platform [2,11,12,16], and 5 (56%) listed other survey software (eg, SurveyMonkey, Research Electronic Data Capture, and the Canadian Fluid Survey System) [13,17,19-21]; the type of web-based system was not reported in 3/12 (25%) studies [10,14,15,18].

The methods used to select indicators for the survey were reported in all 12 studies. The most common method was a literature review (alone or in addition to stakeholder feedback (8/12, 67%) [2,11-13,16,17,20,21]. The number of indicators in the first rating were reported in all 12 studies, and ranged from 6 to 524 items, with a median of 48 items. Only 1/12 (8%) studies reported sending materials to participants prior to the panel sessions and included a document with rationale, methods, and indicator specifications [2]. Duration of the consensus process was reported in 7/12 (58%) studies [2,10,11,13,16,17,21]; median duration was 12 weeks. The geographical scope of expert panel members was reported in all studies; 9 (75%) panels were classified as national [2,10,12,13,16,18-21], and 3 (25%) were classified as international [11,14,15,17].

All studies specified the criteria used to rate each indicator (ie, relevance, importance, feasibility, etc) [2,10-21]. Half of the studies (6/12, 50%) used between one and two types of selection criteria [12,16,17,19-21]; the remaining studies reported using three or more selection criteria [2,10,11,13-15,18]. After the first rating, 5/12 (42%) studies did not report providing feedback of results to panelists [13-15,18-20]. The remaining studies reported providing panelists with frequency distributions, medians, and interquartile ranges for the group, as well as panelists’ own responses compared to the group [2,10-12,16,17,21]. Researchers in 1 (8%) study revised a list of clinical indications based on input from panelists in Rating 1 and distributed this information to participants after the first rating [17]. During this time, panelists review and discuss the ratings, focusing on indications with significant disagreement [1]. However, only 6/12 (50%) of the studies reported included a panelist discussion period after the first round of ratings [2,11,12,16,17,21]; the discussions in 5/6 (83%) studies were either asynchronous, anonymous, or moderated web-based discussions [2,11,12,16,21]; 1 (8%) study used a nonanonymous synchronous webinar format [17].

### Web-Based Expert Panel Results

Most studies (8/12; 67%) reported a second round of ratings [2,10-12,16,18,20,21], with 1/8 (13%) study indicating a third rating was conducted (Table 1) [18]**.** All studies provided information about how indications were selected for the final list. In 8/12 (67%) studies, items were selected following the RAM criteria for disagreement (where the calculated interpercentile range is greater than the interpercentile range adjusted for symmetry, with a panel median score between 6 and 9) [1,2,10-12,16,18,20,21]. The methods used to select indicators differed in the 4 (33%) remaining studies. In the first study, indicators were included based on the following two conditions: (1) the median score for each item was between 8 and 9 and (2) at least 70% of the panelists rated an item in the top third of the scale [17]. In the second study, at least three-quarters of panelists had to agree on an item for it to be selected [14,15]. In the third study, if the average agreement was at least 70% or higher across all 4 criteria of preventability for each item, then the indication was selected [13]. In the last study, more than 50% of panelists had to rate an indicator “extremely important” (ie, 9) for it to be selected [19].

### Web-Based Expert Panel Process Assessments and Satisfaction

Approaches for reporting process assessments and satisfaction were not included in recommendations from Boulkedid et al [9]. In our review, very few studies (3/12, 25%) reported process assessments (eg, level of engagement) or panelist satisfaction (Table 1) [11,14,15,21]. The 3 (25%) studies that did report an assessment of process quality focused on narrowly defined characteristics of satisfaction [11,14,15,21]. For example, one study reported that panelists would have liked more time to discuss ideas in a conference call [11]. In another study, it was reported that most panelists were satisfied with their degree of anonymity throughout the rating rounds [21]. The third study reported most panelists felt the web-based RAM process was “suitable for achieving consensus” [14,15].

## Discussion

### Principal Findings

Web-based RAM panels are increasingly used in health research as an effective, efficient, convenient, and acceptable alternative to traditional consensus processes [22,23]. Previous systematic reviews have assessed the implementation and reporting of the in-person or “modified” Delphi method in research settings [9]. Despite the growing prominence of the virtual RAM, there has been no literature review of design and conduct using completely virtual methods. Documentation, however, is vitally important for researchers to replicate RAM procedures and learn and improve the process across studies. It is also important to assess the validity and applicability of the process and to interpret the results of these studies. Our narrative review of the web-based RAM process in health research helps to fill this gap. Our results show that studies generally provide little information about how the web-based RAM was implemented, making it difficult to interpret and compare study results. After summarizing the main findings of our literature review, we suggest preliminary recommendations for ways to improve the implementation and reporting of virtual RAMs.

The first contribution of this study is to illustrate the underreporting of the web-based RAM process in health research. Our narrative review of 12 unique studies revealed that the vast majority provided only brief descriptions of how their virtual RAM process was implemented. For example, half of the studies did not report a discussion period between rating rounds even though this is a standard feature in the RAM panel process. Adequate time for discussion between rating rounds is necessary for reviewing the distribution of rating results and adjusting the list of clinical indications if necessary. Additionally, although all studies reported the number of panelists who participated in each round, no studies reported consistency of participation across rounds; this is important information to assess the quality and nature of recommendations, ideally generated by highly engaged panel members who consistently participated across the rounds.

Without a common framework for reporting results from web-based RAMs, it is difficult to compare the results across studies. Improved intentionality in designing and transparency in reporting would yield improved results for individual study teams while also allowing external researchers to learn, understand, and build on the process that was used to generate a given set of expert recommendations. Thus, we offer preliminary recommendations for ways the broader field could improve the consistency of the implementation of web-based RAND (RAND Corporation) processes and considerations for individual research teams in designing and reporting on their web-based expert panel studies. We hope these recommendations serve as a launching point for continued development to improve the implementation and reporting of web-based RAMs.

### Preliminary Recommendation 1: Establish Data Collection and Reporting Standards for Web-Based RAM Panels

In the intervening decades since the RAM was developed, this method was continuously refined through its practical application in a wide variety of research settings. It was not until 2001 that the RAND Corporation issued a specific set of recommendations and guidelines for implementing the RAM [1]. However, the availability of this guidance alone does not ensure consistency.

As the systematic review of the Delphi Method by Boulkedid et al [9] revealed, there is still considerable variation in implementation and reporting among research teams using modified versions of the more established method. Thus, we recommend that a professional organization convene a group of experts (eg, journal editors, practitioners, RAM users, etc) to formulate a parallel set of best practices that mirror those developed for the in-person Delphi and Rand/UCLA Appropriateness expert panels. Research teams should clearly describe the data collected and any methodological modifications made to the standard RAM. Multimedia Appendix 3 can be used as a template to report these changes for a single study. Because of word limitations, it may be necessary for researchers to develop a separate protocol paper or to report details in appendices that accompany published findings. In our own work in which we conducted a technology-based RAND expert panel, we reported most of the suggested data elements in an extensive array of supplemental files [24]. Transparent and comprehensive reporting of web-based methods will promote the reproducibility of web-based RAM processes. Based on our own experience, and drawing on results from our literature review, we offer 2 additional recommendations.

### Preliminary Recommendation 2: Establish Measures of Process Quality

We encourage researchers to develop and assess measures of process quality. Process quality can be assessed by asking expert participants to complete a survey at key points throughout the panel process or at its conclusion. Ideally, this would be carried out in the same web-based platform used to host the expert panel. Based on our own experience leading RAND panels, it is feasible to elicit this feedback. This feedback can yield useful quantitative and qualitative data (from numerical ratings and open-text feedback), which researchers can use to refine future rounds and evaluate ongoing processes, as well as using them for future planning purposes [24]. This review found scant reporting of such measures. Table 1 shows the few studies that reported facets of process quality (3/12, 25%), which did so for only narrowly defined assessments, including the need to discuss ideas over a conference call [13], satisfaction with rating anonymity [21], and the suitability of the process for “achieving consensus” [14,15]. Boulkedid et al [9] did not include these types of measures in their systematic review of the Delphi Method. We recommend eliciting and reporting panelist satisfaction as an indicator of process quality. Panel members may rate their satisfaction with all aspects of the RAM process, including the following: (1) background materials provided (if any); (2) process for revising indications; (3) meeting facilitation; (4) the web-based software used; and (5) their likelihood of participating in a similar process again. We also recommend reporting consistency of participation across rounds of the web-based RAM (eg, of the individuals participating in the first round, the percentage who also participated in subsequent rounds, and whether new participants were added across the rounds). Consistency is an important indicator of the depth of commitment and depth of thought as individuals consider and reconsider ratings across multiple rounds.

### Preliminary Recommendation 3: Consider Other Viable Technical Platforms With Similar Levels of Functionality

Most studies included in our review used a web-based platform to conduct RAND panels. Although ExpertLens (RAND Corporation) was the most often used web-based platform among the studies in our review, our own experience using Group System’s ThinkTank suggests there are other platforms with similar or perhaps expanded levels of functionality that could be considered [24,25]. A web-based hosting platform should have the following functions: (1) allow teams to engage, collaborate with, and capture and organize input from a large number of individuals; (2) allow for sharing, revising, organizing, and analyzing content in real time or asynchronously; and (3) allow teams to export session content (eg, ratings) to formats such as Microsoft Excel for further analysis.

### Limitations

Although our review identified gaps in the literature, there are limitations. The PubMed database does not search full text, so we may have missed articles that reference using web-based RAM in the main text, but not in the title or abstract. This may have limited the number of articles included in our review. In addition, our article selection criteria were narrow, which again could have limited the number of articles included in our review. We also acknowledge that the time frame for our literature search stopped just before the start of the COVID-19 pandemic. Future work should include conducting an updated review, perhaps applying our preliminary recommendations to assess reporting in more recent studies. This study is, to our knowledge, the only investigation to formally review and summarize the literature on using completely web-based RAM approaches. We recommend further development of more formal reporting standards for running web-based RAM panels. Web-based approaches have undoubtedly grown during the pandemic as more and more research has moved to internet-based platforms, a trend that is likely to remain. Our preliminary reporting recommendations may encourage other researchers to report these details to increase research transparency and replicability. This methodological transparency is important for building and expanding knowledge of best practices for conducting RAMs virtually.

### Conclusion

In conclusion, conducting research virtually has become particularly important within the context of the COVID-19 pandemic due to the prohibitions and safety concerns about in-person group meetings. This shift to web-based workplaces may outlast the pandemic [26]. We also anticipate that the multiple benefits associated with web-based collaboration will make the RAM with a web-based component an appealing and cost-effective option for researchers seeking an efficient process for incorporating expert opinion into developing recommendations. This narrative review reveals underreported yet important characteristics for conducting and reporting on web-based RAM expert panels. Without a common framework for reporting results from web-based RAMs, it is difficult to compare results across studies. In this way, intentionality in designing and transparency in reporting will yield improved results for individual study teams while also allowing external researchers to understand the process that was used to generate a given set of expert recommendations. We highlight preliminary recommendations for conducting and evaluating virtual RAM approaches that will contribute to replicable high-quality findings using web-based RAMs.

## Appendix

Multimedia Appendix 1 Literature search strategy.

Multimedia Appendix 2 Criteria for literature search.

Multimedia Appendix 3 Summaries of articles included in literature review.

## Abbreviations

RAM: RAND/UCLA Appropriateness Method
