# Supplementary material for: Planning and Reporting Effective Web-Based RAND/UCLA Appropriateness Method Panels: Literature Review and Preliminary Recommendations
Source: J Med Internet Res. 2022 Aug 26;24(8):e33898. doi: 10.2196/33898 (PMC9463617; doi:10.2196/33898)
Supplement: Multimedia Appendix 1 [file jmir_v24i8e33898_app1.pdf]

Multimedia Appendix

Literature search strategy.

Search Strategy	Number of Articles	Database	Search Details	Notes
((RAND/UCLA Appropriateness Method*) OR (RAND Appropriateness Method*) OR (modified RAND) OR (RAND AND panel)) AND (online OR e-Delphi OR web OR virtual))	78	PubMed	((rand/ucla appropriateness method[All Fields] OR rand/ucla appropriateness methodology[All Fields]) OR (rand appropriateness method[All Fields] OR rand appropriateness methodology[All Fields]) OR (modified[All Fields] AND RAND[All Fields]) OR (RAND[All Fields] AND panel[All Fields])) AND (online[All Fields] OR e-Delphi[All Fields] OR web[All Fields] OR virtual[All Fields])	Date of search: 03/01/2019 (saved in PubMed)<br><br>Results with filters:<br>1) Publication date = 10 years<br>2) Languages = English
