# Supplementary material for: Planning and Reporting Effective Web-Based RAND/UCLA Appropriateness Method Panels: Literature Review and Preliminary Recommendations
Source: J Med Internet Res. 2022 Aug 26;24(8):e33898. doi: 10.2196/33898 (PMC9463617; doi:10.2196/33898)
Supplement: Multimedia Appendix 2 [file jmir_v24i8e33898_app2.pdf]

Multimedia Appendix

Criteria for literature search.

**Inclusion Criteria**
Published in English
Published between 2009-2019
Paper focuses on at least one of the three topic areas:<br>a. Performance indicators for medical care<br>b. Informing clinical guidelines for diagnosis, treatment or screening<br>c. Informing the implementation of clinical guidelines
Used RAND/UCLA Appropriateness Method to measure the appropriateness of a medical procedure
Expert panel rating was conducted online-only
**Exclusion Criteria**
Not published in English
Not published between 2009-2019
Does not focus on any of the following:<br>a. Performance indicators for medical care<br>b. Informing clinical guidelines<br>c. Informing the implementation of clinical guidelines
Not geared towards providers
Systematic review/summary of the literature
Not online-only
Did not use RAND/UCLA Appropriateness Method to measure the appropriateness of a medical procedure
